# Humpback whale genomes reflect the increased efficiency of commercial whaling

**DOI:** 10.1126/sciadv.ady7091

**Published:** 2025-12-17

**Authors:** Fabricio Furni, Martine Bérubé, Marcos Suárez-Menéndez, Eduardo R. Secchi, Randall R. Reeves, Jooke Robbins, Camilla F. Speller, Per J. Palsbøll

**Affiliations:** ^1^ Groningen Institute for Evolutionary Life Sciences, University of Groningen, Groningen, Netherlands.; ^2^ Center for Coastal Studies, Provincetown, MA, USA.; ^3^ Institute of Oceanography, Federal University of Rio Grande, Rio Grande, Brazil.; ^4^ Okapi Wildlife Associates, Quebec, Canada.; ^5^ Department of Anthropology, University of British Columbia, Vancouver, British Columbia, Canada.

## Abstract

Genetic diversity is declining globally, a trend that may particularly affect exploited populations that must adapt to rapid environmental change and other threats. Estimated genomic changes in effective population size mirrored known whaling history and shifts in technology. In the Southern Ocean, a comparison of genomes from historical and contemporary populations indicated that the contemporary genomes have less diversity and an elevated realized mutation load for moderately deleterious mutations, likely due to the effects of whaling. Our results demonstrate that the relatively recent, brief, and marked depletion of humpback whale populations by whaling likely had subtle but discernible, negative, and lasting effects on the whales’ genomes. Thus, even as some humpback whale populations are now recovering to pre-exploitation numbers, they likely do so with a diminished adaptive capacity in the face of future conditions and threats.

## Teaser

Humpback whale genomes reflect shifts in whaling practices and their potential impact on the animals’ adaptive capacity.

## INTRODUCTION

The well-documented, ongoing, and accelerated anthropogenic loss of biodiversity (*1*) threatens the integrity of ecosystems globally and, ultimately, human welfare. However, the effect on genetic diversity, the very basis of biodiversity, remains elusive. The genetic makeup of conspecific individuals ultimately determines their population’s and species’ long-term persistence as well as their ability to adapt to environmental change, such as global warming. A recent meta-analysis by Shaw *et al.* (*2*) revealed a decline in genetic diversity in 628 species across a wide array of environments, suggesting an ongoing global loss of genetic diversity. Those authors inferred that this widespread loss of genetic diversity was caused by the increase in anthropogenic pressures over the period covered by the reviewed studies. However, it remains largely unknown how much of the loss can be attributed to anthropogenic pressures and if this recent, and possibly transient, loss affects individual fitness.

The rate of loss of genetic diversity is reciprocally related to the effective population size (*N*_E_). Populations with a large *N*_E_ are subject to low rates of random genetic drift, resulting in higher levels of genetic diversity and comparably more efficient selection against detrimental traits (*3*). In contrast, the effects of genetic drift dominate at low *N*_E_, resulting in an elevated rate of random changes in the frequencies of genetic variants, potentially leading to loss of adaptive traits, i.e., genetic erosion (*4*, *5*). The random changes in variant frequencies may supersede natural selection, thus minimizing selection against detrimental variants. Consequently, small, inbred populations may suffer from lowered average fitness due to an increase in mutation load (*4*, *6*, *7*), ultimately increasing the risk of population extinction (*4*, *8*). Several empirical studies have indicated mutation load in wild contexts (*9*–*12*), including human-induced bottlenecked populations, such as those of the northern elephant seal (*Mirounga angustirostris*) (*13*), Grauer’s gorilla (*Gorilla beringei graueri*) (*14*), Scandinavian gray wolf (*Canis lupus*) (*15*), and the whooping crane (*Grus americana*) (*16*). Luckily, theoretical advances have enabled detailed characterizations of recent changes in *N*_E_ (i.e., as far back as ~200 generations) (*17*). This facilitates the quantitative assessment of the relative impact of recent anthropogenic pressures on *N*_E_ in wild populations and of changes in mutation load.

Commercial whaling of large whales during the late 19th and early 20th centuries is a textbook example of recent overexploitation, which brought most populations of large whales to the brink of extinction (*18*). Although large knowledge gaps remain, the exploitation history of humpback whales, *Megaptera novaeangliae*, is comparatively well documented, providing an excellent opportunity to assess the impacts of whaling on *N*_E_ and genetic erosion. Humpback whales became a primary target of commercial whaling during the mid-19th century after the slower right and bowhead whales (Balaenidae spp.) had been depleted (*19*). Much of the early humpback whaling was conducted from shore stations in coastal waters but later expanded into pelagic waters, as whales became scarce locally (*19*). The adoption of faster, steam-propelled ships and rocket-propelled explosive harpoons during the latter half of the 19th century marked the beginning of an era of highly efficient mechanized, industrial-scale whaling. This era drove the already depleted humpback whale to the brink of extinction in the North Atlantic by the early 20th century (*20*–*24*). Consequently, whaling companies shifted their focus to distant, but abundant, whale populations in the Southern Ocean. Shore whaling stations established in Antarctica early in the 20th century ushered in a period of mechanized whaling at an unprecedented scale (*18*). As a result, Southern Ocean humpback whale populations were reduced to very low levels within a few decades (*18*, *25*). As the whalers moved offshore using an efficient combination of mechanized factory ships and catcher boats, they targeted other large pelagic whales and humpback whales. The rapid depletion of large whales by the whaling industry prompted the introduction of progressively stronger regulatory measures during the 1930s, eventually leading to the formation of the International Whaling Commission in 1946. The Commission banned commercial whaling for humpback whales in the Southern Ocean in 1963. However, despite this internationally agreed-upon ban, the former Soviet Union whaling fleet continued to covertly hunt humpback whales, killing an additional 46,000 humpback whales in the Southern Ocean (*26*, *27*).

**Fig. 1.**
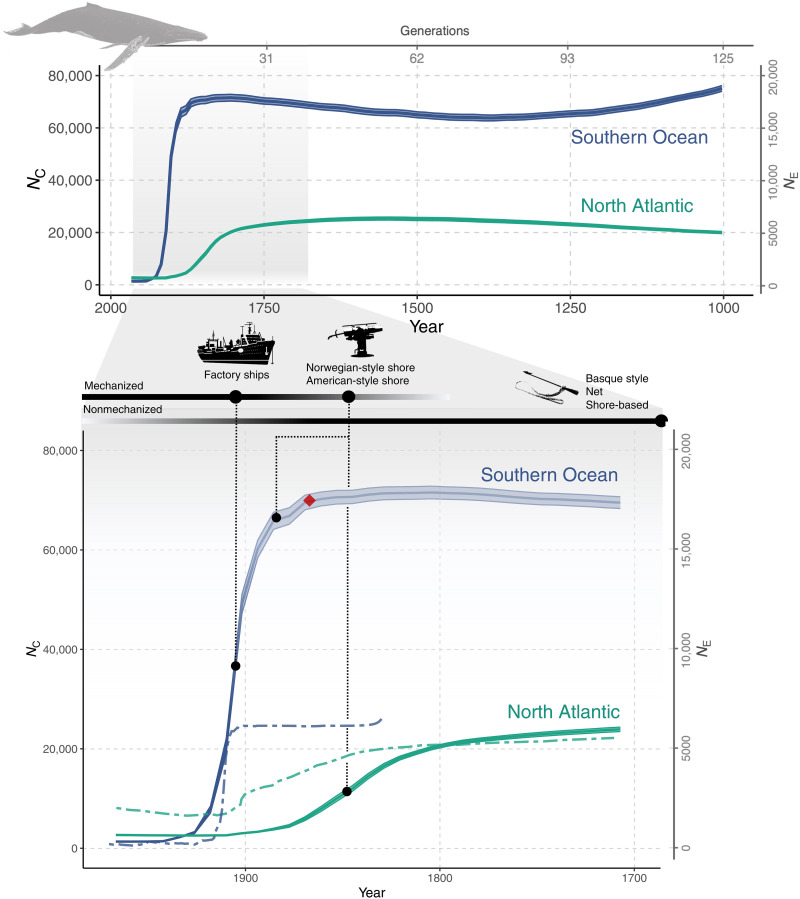
Inferred historical abundance of humpback whales in the western North Atlantic (green) and Southern Ocean (blue). *N*_C_ values were derived from the linkage disequilibrium–based *N*_E_ estimates using a *N*_E_:*N*_C_ ratio of 1:4 (*34*); the red diamond denotes the calibration point used to estimate the postwhaling average number of calendar years per generation. Whaling periods and major changes in whaling technology (*94*) are noted along the top horizontal axis in the bottom panel. Modeling-based estimates of *N*_C_ in the Southern Hemisphere (blue dashed line) and North Atlantic (green dashed line) were obtained from Zerbini *et al.* (*25*) and Punt *et al.* (*20*), respectively.

The effects of whaling on humpback whale abundance have been inferred using demographic population modeling (*20*, *25*), which has mirrored what was known about the history of humpback whaling. The modeling results from the North Atlantic estimated a gradual decline in humpback whale abundance beginning in the early to mid-19th century and reaching a low point during the early 20th century (Fig. 1). Similar assessments for the Southern Ocean inferred a high prewhaling abundance of humpback whales, which was relatively stable until the turn of the 20th century when shore-based mechanized whaling in the Southern Ocean started. The first two to three decades of the 20th century were marked by a marked decline, after which humpback whale abundance remained very low (Fig. 1). In both oceans, humpback whales now show signs of recovery in most areas (*25*, *28*, *29*).

Here, we took advantage of the difference in whaling histories between the North Atlantic and the Southern Ocean, which together reflected the evolution of commercial whaling over time, to assess the genomic effects on humpback whale populations. To this end, we analyzed whole-genome DNA sequences from 16 contemporary (1980 and onward) and 9 historical (early 20th century) humpback whales.

## RESULTS AND DISCUSSION

### Changes in whaling practices are reflected in the genomes of humpback whales

We estimated past changes in *N*_E_ from the degree of linkage observed in contemporary humpback whale genomes (Fig. 1 and fig. S1). The estimated history of changes in humpback whale abundances in the Southern Ocean and North Atlantic closely mirrored the known whaling history and trends in abundance inferred in population modeling–based studies (*20*, *25*). The well-known marked reduction in humpback whale abundance in the Southern Ocean during the first three decades of the 20th century from mechanized whaling (*18*) was evident in our estimation, which placed the whaling bottleneck at 13 to 14 generations ago. This single event provided us with a temporal calibration point akin to fossil records or geological events used in dating species splits in DNA-based phylogenies (*30*). From this observation, we calculated an average postwhaling bottleneck generation time of 8 years, which we used to convert our genome-based temporal estimates in generations into calendar years. A generation time of 8 years is considerably lower than earlier estimates of the generation time in humpback whales, which ranged from 14.1 to 25.5 years (*31*). However, generation time in long-lived mammals is not a constant entity but varies with the underlying abundance, population growth rate, and even anthropogenic pressures. For instance, a growing population far below carrying capacity is expected to have a shorter generation time compared to a stable population near or at carrying capacity (*32*, *33*). Similarly, whalers may have targeted larger, older whales, possibly further depressing the average generation time. The severe depletion of Southern Ocean humpback whales in the early to mid-20th century by mechanized whaling and later by extensive illegal whaling by the former Soviet Union (*27*) kept the abundance low and thus likely the generation time short for a considerable period. In the same vein, the generation time was likely longer in humpback whale populations before the start of mechanized whaling. Hence, the calendar year estimates reported here that predate whaling are likely underestimated. However, since our estimates of the historical abundances showed relatively constant population sizes, the effect of a longer generation time on our estimate of prewhaling abundances should be negligible. The estimation of “time” is also dependent on the generational recombination rate, which has not been estimated in cetaceans.

We used a ratio of 1:4 for *N*_E_ to census population size (*N*_C_) (*34*), a value used in earlier studies of baleen whales (*34*–*37*). Using this value, the historical abundance of North Atlantic humpback whales before mechanized whaling, around the year ~1600, was estimated at 25,184 individuals [95% confidence interval (CI), 24,616 to 25,732; Fig. 1], which began to decline around the year 1800. The subsequent, linear, continuous drop in *N*_C_ (Fig. 1) in the North Atlantic eventually stabilized at its lowest level at the turn of the 20th century (Fig. 1), where we observed the lowest humpback whale abundance in the North Atlantic around the year 1900 at 2592 (95% CI, 2568 to 2620; Fig. 1). These estimates are consistent with estimates based on population modeling [e.g., prewhaling abundance from 17,151 to 22,647 (*18*); Fig. 1] and genetic diversity based on pedigree mutation rates [at ~20,000 (*35*)]. The observed continuous decline in *N*_C_ coincided with the expansion of humpback whaling efforts, i.e., the whalers moved further offshore, adopting more efficient technologies. The most recent estimate of abundance for North Atlantic humpback whales (1992–1993) was 10,752 (*29*).

When humpback whales became scarce in the North Atlantic during the 19th century, the beginning of a declining trend in *N*_E_ was observed in the Southern Ocean humpback whale genomes. This initial 19th-century decline in the Southern Ocean likely reflected whaling from land-based stations at lower latitudes (*25*). The decline in *N*_E_ in the Southern Ocean quickly accelerated at the start of the 20th century (12 to 13 generations ago) when Northern Hemisphere whalers moved to the Southern Ocean, where whales were more plentiful, marking the dawn of large-scale, mechanized whaling (*18*). A few decades later, around the year 1930, *N*_E_ in the Southern Ocean reached very low levels and continued to decrease but at a slower rate. Our premechanized whaling (year ~1600) estimate of *N*_C_ for Southern Ocean humpback whales was 66,871 (95% CI, 65,672 to 68,959; Fig. 1), and the lowest abundance was detected in the year 1930 at 1375 (95% CI, 1323 to 1426; Fig. 1). Accordingly, the abundance of humpback whales in the Southern Ocean was reduced to 2 to 3% of the premechanized whaling level of abundance during only four generations, illustrating the devastating impacts of mechanized, highly efficient industrial whaling (*18*, *25*). Humpback whales that summer off the western Antarctic Peninsula winter primarily in the eastern South Pacific (*38*, *39*), where recent assessments (*40*, *41*) estimated the contemporary abundance at ~22,000, three times lower than our premechanized whaling abundance estimate. However, our estimate of the premechanized whaling *N*_C_ in the Southern Ocean likely does not reflect the abundance of humpback whales in any specific region but instead the overall “genetic” population, and hence our estimate must be interpreted accordingly. Historically, the more abundant populations in the Southern Ocean were likely more connected, which is reflected in our estimate of the abundance in the Southern Ocean (*38*, *42*–*44*). We note that the *N*_E_:*N*_C_ ratio may vary with changes in demography and life history traits across space and time (*45*, *46*). Differences between the abundances reported here and in previous studies could therefore also, in part, reflect this underlying variation. A better understanding of how and to what extent the *N*_E_:*N*_C_ ratio is affected by these aspects could improve the conversion of *N*_E_ into historical abundance. In addition, the *G* statistic (a measure of the robustness of the estimates of *N*_E_; fig. S2) for the prewhaling abundance was close to 50, suggesting that the Southern Ocean prewhaling estimates were less reliable. The observed loss of accuracy at higher abundances is a well-known feature of linkage-based estimations of *N*_E_ and a consequence of the reciprocal relationship between the degree of linkage and *N*_E_, which is the basis of this approach (*17*). In contrast, the values of the *G* statistic for the *N*_E_ estimates during the periods of commercial and mechanized whaling, as well as the pre-1850s North Atlantic, were at levels suggesting robustness.

The inferred changes in *N*_C_ correlated with the estimated humpback whale catches (fig. S3). In the Southern Ocean and North Atlantic, the cumulative catches explained 68 and 85%, respectively, of the estimated reduction in *N*_C_ (Pearson’s correlation, *r*^2^ = 0.68 and 0.85, *P* < 0.0001). Although correlation does not equal causation, these results align well with the notion that whaling accounted for marked reductions in humpback whale abundances since the initiation of large-scale commercial whaling.

### The genomic consequences of humpback whaling

Severe and prolonged declines in abundance (*N*_E_ specifically) can lead to genomic erosion from an elevation in the rate of random genetic drift and inbreeding (*4*, *47*), resulting in the loss of genetic diversity, including adaptive traits, and possibly an increase in detrimental traits and consequently mutation load (*11*, *48*, *49*). To assess the effect of the marked, but recent and relatively brief, population declines due to whaling on the contemporary humpback whale genomes, we compared humpback whale genomes obtained from bone remains (*N* = 8) collected at the former Grytviken whaling station in South Georgia with the contemporary Southern Ocean humpback whale genomes collected in the waters off the western Antarctic Peninsula. The Grytviken bone samples are from humpback whales caught during the first phase of mechanized whaling in the Southern Ocean (*50*). Accordingly, the bone and contemporary genomes can be viewed as representing the “pre-” and “post-” mechanized commercial whaling populations, respectively. Research on contemporary humpback whales suggests that these whales spend their summers off the western Antarctic Peninsula and South Georgia, as well as their winters in the Southeast Pacific and Southwest Atlantic, respectively. However, multiple lines of evidence have demonstrated connectivity between these two areas [e.g., (*42*–*44*)], which was likely higher between the much larger historical populations. Our clustering results indicate a proximity between historical and contemporary samples, with the contemporary genomes lying near the broader historical population (fig. S4).

**Fig. 2.**
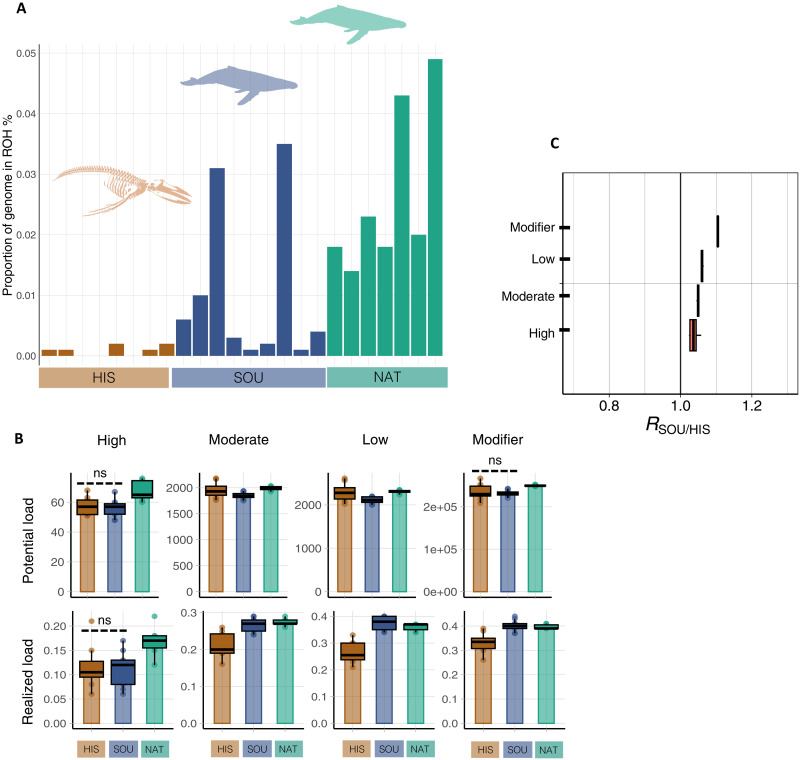
Genomic footprints of whaling in the humpback whale genomes. (**A**) The proportion of the genome in ROHs_> 1 Mbp_. The bars denote each genome. (**B**) Derived variants categorized by inferred impact (high, moderate, low, and modifier). Potential mutation load denotes the counts of derived variants, and realized load denotes the proportion of derived variants in homozygosity. ns, not significant. (**C**) *R_x/y_* ratios between historical and contemporary Southern Ocean genomes (*R*_SOU/HIS_). Note that the estimates obtained from the historical Southern Ocean genomes (HIS), the contemporary Southern Ocean (SOU), and the North Atlantic (NAT) are depicted in brown, blue, and green, respectively.

Given the relatively brief duration (<12 to 14 generations) of the reduction in abundance due to whaling and a minimum *N*_E_ at ~370 (in the Southern Ocean) and ~670 (in the North Atlantic Ocean), the expected decrease in genetic diversity and increase in mutation load should be negligible. The genome-wide heterozygosity in the contemporary Southern Ocean genomes (7.48 × 10^−5^, SD = 4.9 × 10^−6^) was 20 to 30% less than in the historical genomes (10.26 × 10^−5^, SD = 1.7 × 10^−5^) but closer to the heterozygosity in the contemporary western North Atlantic genomes (8.0 × 10^−5^, SD = 1.4 × 10^−6^). The degree of inbreeding is negatively correlated with *N*_E_, which leads to an increase in the fraction of the genome in homozygosity, here captured as runs of homozygosity (ROHs) (*51*). The mean inbreeding coefficient [*F*_ROH > 1 Mbp_, fraction of the genome with ROHs longer than 1 mega–base pair (Mbp)] was lower among the historical genomes (0.0001, SD = 0.0007) than the contemporary genomes in the Southern Ocean (0.014, SD = 0.01). The highest mean *F*_ROH > 1 Mbp_ was detected among the North Atlantic genomes at 0.04 (SD = 0.01; Fig. 2A). Despite an elevated mean *F*_ROH > 1 Mbp_ in both contemporary humpback populations (relative to the historical samples), the observed mean *F*_ROH > 1 Mbp_ values were relatively low and nothing akin to *F*_ROH > 1 Mbp_ estimates reported in very small, more inbred cetacean populations, such as eastern North Pacific “resident” killer whales, *Orcinus orca* (*F*_ROH > 1 Mbp_, 0.18 to 0.44) (*52*) and Gulf of California fin whales, *Balaenoptera physalus* (*F*_ROH > 1 Mbp_, 0.17 to 0.22) (*49*). These results suggest that the brevity of the postwhaling bottleneck, combined with *N*_E_ values between 400 and 800, did not lead to high levels of inbreeding or a marked loss of genome-wide diversity. In addition, changes in structure and connectivity among the large historical and small contemporary populations likely also contributed to the observed “loss” in heterozygosity and increase in *F*_ROH > 1 Mbp_.

A marked, rapid reduction in *N*_E_ to low levels is expected to result in random losses and gains in the frequency of detrimental variants due to comparatively higher rates of genetic drift (*4*, *47*). An increase in the frequency of detrimental variants can subsequently lead to a decline in average fitness, in the short term by exposure of recessive deleterious alleles in offspring of inbred parents and in the longer term by loss of adaptive potential (*53*). We annotated variants and classified them into the following four categories based on their predicted effect on gene function: high (e.g., loss of gene function), moderate (e.g., missense variants), low (e.g., synonymous substitution), and modifier (e.g., no impact) (*54*). For detrimental variants, we detected an overall loss (potential mutation load; Fig. 2B) and an increase in homozygosity (realized mutation load; Fig. 2, B and C) between the historical and contemporary genome sequences across all impact categories except for high-impact variants. The observed direction of change (Fig. 2C) was consistent with an overall loss of genetic variation and a greater variance in variant frequencies due to an elevated rate of genetic drift at a low *N*_E_. In contrast, we did not observe a similar change in either the potential or the realized mutation load among the high-impact variants. In this category, we observed no significant increase in the realized mutation load between the historical and contemporary Southern Ocean genomes (two-tailed Mann-Whitney *U* test, *P* = 0.56). The impact category assigned to a variant is a reflection of its likely phenotypic effect and, hence, the degree of selection against the specific variant (*5*). The degree of random changes in population frequencies at an *N*_E_ of 400 to 800 due to genetic drift will be relatively modest, implying that the efficacy of selection remains high for high-impact variants but less so for modest- and low-impact variants (*12*, *16*, *55*), as observed in our data. The temporal changes in allele frequencies for the four impact categories between the historical and contemporary Southern Ocean genomes were estimated as the *R*_*x/y*_ ratio (*56*). *R*_SOU/HIS_ estimates were above one, irrespective of impact category, suggesting a slight increase in moderately detrimental alleles among the contemporary Southern Ocean genomes.

Overall, we observed reduced levels of genetic diversity, a modest increase in inbreeding, and a higher realized mutation load for mildly deleterious variants in the contemporary versus the historical genomes of the Southern Ocean. The same outcome was confirmed when using only transversion variants, which are less affected by deamination artifacts (fig. S5 and Supplementary Text). We also performed forward simulations (*57*), which supported the trends observed in our Southern Ocean results, although the magnitude of simulated changes was smaller (fig. S6). For instance, simulated genome-wide heterozygosity loss ranged from 2 to 12%, compared with 20 to 30% in the empirical data. Nevertheless, the simulations mirrored the modest accumulation of mildly deleterious variants and an increase in ROHs. The difference in genome-wide heterozygosity and mutation load between the historical and contemporary genomes in the Southern Ocean is likely the outcome of multiple factors, among which mechanized commercial whaling likely was a main contributor. Spatiotemporal changes in population connectivity (also due to whaling) may also have contributed to the observed differences [e.g., (*42*–*44*)]. Although some connectivity among contemporary populations has been reported (*42*), the degree of genetic connectivity (i.e., the number of migrants per generation) has likely decreased because of population reductions. These temporal differences in population continuity can affect the observed differences between the contemporary and historical samples. As in this study, depleted populations in other wild species have also been subjected to similar levels of genomic erosion despite a recent recovery (*13*, *16*, *58*). For instance, Grauer’s gorillas underwent a rapid human-induced bottleneck to ~4000 individuals over 20 years, resulting in a 20% decline in genome-wide heterozygosity and an increase in mutational load (*14*). Similarly, the southern white rhinoceros (*Ceratotherium simum*) declined to ~200 individuals during the early 20th century due to hunting and habitat loss. A comparison of historical and contemporary genomes revealed a 36% loss of heterozygosity (*59*). Collectively, these studies support the level of loss in genome-wide heterozygosity observed here because of a recent, marked, and sudden human-induced bottleneck.

The standing genetic diversity in a population constitutes the raw material upon which natural selection can act, potentially leading to adaptations that affect the short- and long-term fate of wild populations. From a conservation perspective, the main concerns are the processes that result in a low *N*_E_, when the efficacy of selection is greatly diminished and random genetic drift dominates, potentially leading to inbreeding depression and loss of adaptive potential, thereby increasing the risk of extinction (*4*, *60*, *61*). Here, we document a marked reduction in *N*_E_ from mechanized whaling to an *N*_E_ at ~370 after only a few decades of humpback whaling in the Southern Ocean. A similar reduction (to ~670) was observed in the North Atlantic after at least a century of increasingly efficient whaling. Although the populations in both ocean basins appear to be recovering in most areas (*28*, *29*, *62*) and the low-point *N*_E_ values were at levels where the effects of random genetic drift are expected to be modest, we did observe lower genetic diversity and an elevated realized mutation load in the contemporary populations. The temporal changes in abundance estimated in our study that presumably led to these effects correlated highly with the known whaling catch records. Although these correlations do not prove causation, in all likelihood, mechanized commercial whaling contributed substantially to the severe reductions in abundance. Our estimated temporal trends in humpback whale abundance mirrored the trends that have emerged from population modeling, which was based on whaling data only.

The extent of the observed genomic changes on the species’ fitness is difficult to predict but may affect humpback whales’ ability to adapt to current and future ecosystem change, thus warranting continued monitoring and assessment of aspects such as heterozygosity and mutation load (*4*, *13*, *60*, *63*, *64*). The genomic effects we detected here were in the current populations, i.e., the immediate postwhaling generations. Consequently, the full impact of the increases in inbreeding and mutation load for moderate-impact variants remains unknown and may have long-term consequences persisting well after populations have recovered. In light of current anthropogenic pressures, further assessments on the fitness effect of the observed levels of detrimental variants are necessary to understand the long-term impacts of whaling on the species.

On a more positive note, combining coalescent theory and genome linkage in an analysis based on a relatively modest sample size had sufficient power to accurately capture population size changes during the most recent 10 to 15 generations. The commercial nature of most mechanized whaling required detailed recordkeeping of catches and, hence, a comparatively well-characterized history of human exploitation (*18*, *19*). This is not the case for many other exploited species. The consistency of our results with historical records suggests great potential for similar analyses to provide independent and detailed insights into the effects of past exploitation or other anthropogenic pressures on other species to aid in their conservation.

## MATERIALS AND METHODS

### Sample collection and identification

Historical bone samples (*N* = 12) were collected from the area surrounding the former whaling station in Grytviken, South Georgia Island. Approximately 4 cm^2^ of bone samples were collected from individual skulls. Contemporary humpback whale, *M. novaeangliae*, tissue samples were collected as skin biopsy samples from free-ranging humpback whales (*65*) off the western Antarctic Peninsula (Southern Ocean; *N* = 9) and in the Gulf of Maine (western North Atlantic; *N* = 7).

### Species identification of bone remains

Peptide mass fingerprinting of bone collagen was used to identify the historical bone samples to species level (*66*, *67*). Approximately 10 to 30 mg of bone were demineralized in 250 μl of 0.6 M HCl at 4°C. Once demineralized, samples were centrifuged, the supernatant was discarded, and the remainder was rinsed with 250 μl of 0.1 M NaOH, followed by three rinses each with 250 μl of 50 mM ammonium bicarbonate (AmBic) (pH 8.0). The extracted collagen was gelatinized in 100 μl of AmBic solution at 65°C for 1 hour, and 50 μl of the resulting supernatant was incubated overnight with 0.4 μg of trypsin at 37°C. Trypsin was deactivated by the addition of 1 μl of 5% trifluoroacetic acid and subsequently purified with 100 μl of C18 resin ZipTip pipette tips (EMD Millipore Inc.). Next, 1 μl of extracted collagen was spotted in triplicate with 1 μl of matrix solution (α-cyanno-4-hydroxycinnamic acid) along with calibration standards on a 384-spot matrix-assisted laser desorption/ionization (MALDI) target plate and run on an Ultraflex III MALDI time-of-flight (TOF)/TOF mass spectrometer (Bruker Inc.). Spectral replicates were averaged using the software MMASS (v. 3) (*68*) and compared to the list of mass/charge ratio markers for marine mammals presented in (*66*, *69*, *70*). Once taxonomically identified, a subset of 16 humpback whale bones was selected for further genomic analysis.

### DNA extraction and genome sequencing

Genomic DNA was extracted from 16 historical bones following the strict protocols for ancient DNA in the dedicated clean laboratory facilities of the Ancient DNA and Proteins (ADαPT) Facility at the University of British Columbia (Vancouver, British Columbia, Canada). Decontamination was conducted by immersing ~200 mg of bone samples in a 6% sodium hypochlorite solution for 5 min, subsequently rinsing twice with ultrapure water, and exposing them to ultraviolet radiation for 20 min on two sides. Bone fragments were powdered, subjected to a 1-hour predigestion step (*71*), and then fully digested overnight at 50°C in 3 ml of lysis buffer [0.5 M EDTA and Proteinase K (0.5 mg/ml). Extraction of genomic DNA was conducted using the column filter protocol described by Yang *et al.* (*72*) and modified as in Yang *et al.* (*72*). Genomic libraries were generated using the dual-index double-strand protocol described by Meyer and Kircher (*73*). Extraction and library blank controls were added to each for contamination control. Library quality was checked using a BioAnalyzer (Agilent Inc.) and quantitative polymerase chain reaction. An initial screening of the endogenous DNA content was performed by sequencing equimolar-pooled libraries in a single lane on a HiSeq 2500 (Illumina Inc., USA) at 150 cycles, single-end sequencing in rapid mode. After the initial screening, 12 samples with the highest endogenous DNA content were pooled and paired-end sequenced in an S4 flow cell on the NovaSeq 6000 (Illumina Inc., USA) at 150 cycles.

The Gentra Puregene Tissue Kit (QIAGEN Inc.) was used following the manufacturer’s protocol to extract genomic DNA from contemporary skin biopsy samples at the Marine Evolution and Conservation laboratory, University of Groningen, Netherlands. Genomic DNA was resuspended in 1× TE buffer [10 mM tris-HCl and 1 mM EDTA (pH 8.0)]. The concentration of DNA was determined by fluorometric quantification using a Qubit 2.0 Fluorometer (Life Technologies Inc.) and the broad-range protocol. The molecular weight of the extracted DNA was assessed by gel electrophoresis through a 0.7% agarose matrix stained with ethidium bromide at 175 V for ~30 min. Whole-genome resequencing was conducted as DNBseq, 100 cycles, paired-end on a BGISEQ500 platform (BGI Inc.) by the Beijing Genomics Institute, Europe. Genomic sequences for the Gulf of Maine humpback whales were retrieved from the National Center for Biotechnology Information (NCBI) BioProject PRJNA990679 (*35*).

### Genome mapping and variant calling

Raw reads from historical bone samples were trimmed at the first 10 bp at both ends using CUTADAPT (v. 5.0) (*74*) due to deamination commonly present in ancient DNA (*75*). For contemporary biopsy samples, raw reads were not trimmed at the ends and aligned, sorted, and had duplicates removed as described above. An additional step was performed for the historical samples to reduce DNA damage patterns from the dataset. In this additional step, the damage was estimated, and if present, the read alignment quality was recalibrated to lower values (<10) using mapDamage (v. 2.0) (*76*). Variants were called using GATK (v. 4.1) (*77*). Genotypes were called using GATK HaplotypeCaller in genomic variant call format (GVCF) mode per chromosome, excluding reads with a mapping quality of <30. Samples were merged with GATK CombineGVCFs, and joint genotype calls were performed with GATK GenotypeGVCF, including nonvariant sites. Variant sites were initially filtered by quality (QUAL > 40), missing data (“F_MISSING < 0.001”), indels and nonbiallelic single-nucleotide polymorphisms (SNPs) removed (-M 2 -m2 -–type snps), and data from nonautosomal chromosomes (−t ^NC_045806.1, ^NC_045807.1, ^NC_001601.1) with BCFtools view (v. 1.15) (*78*). Individual genotypes were filtered by a minimum depth of 6× and minimum genotype quality at 15 [-i “MIN(FMT/DP)>5 & MIN(FMT/GQ)>15”] in the contemporary datasets.

For temporal comparisons (e.g., genetic load), historical samples were merged with contemporary samples in a “temporal dataset.” To merge the dataset, the average sequencing depth was normalized by downsampling high-depth contemporary samples to the average sequencing depth at 6× observed in the historical bone samples (tables S1 and S2). Downsampling was performed on BAM files using SAMtools view, applying a random fraction (-s) from the overall data to achieve an average sequencing depth of 6× (table S1). Joint variant calling and initial filtering steps were performed as described above. In addition, a customized script was applied to filter individual genotypes by a minimum sequencing depth of 3× for the variant allele, and in the case of heterozygous genotypes, a Mendelian balance between 0.35 and 0.75 (if not in the Mendelian balance, the genotype was considered as homozygous for the most common allele). Sites containing low-frequency transition variants (variant allele count < 3) were also excluded from the dataset since they most likely reflect deamination artifacts in the DNA obtained from the historical samples.

### Historical DNA authentication

For historical DNA authentication, raw reads were not trimmed at the ends (as the ends hold most of the deaminated sites) and mapped against the blue whale, *Balaenoptera musculus*, reference genome (NCBI GenBank accession GCA_009873245.3) (*79*) using BWA mem (v. 0.7) (*78*). Aligned reads were sorted, duplicates were marked and removed, and alignment metrics were collected using Picard (v. 2.22) (*80*). Postmortem DNA damage was estimated using mapDamage (v. 2.0) (*76*).

### Functional annotation of variants

SnpEff (v. 5.2) (*54*) annotates variants based on the most likely effect of the substitution on the downstream translation, classifying variants as high (e.g., loss of function and stop loss), moderate (e.g., missense mutations), low (e.g., synonymous variants), and modifier (e.g., no impact). The ancestral state of each variant was inferred as the state shared with either fin whales, *B. physalus*, or blue whales, *B. musculus*. We merged a fin whale genome (NCBI GenBank accession SAMN43059233) (*81*) and the blue whale reference genome sequence (NCBI GenBank accession GCA_009873245.3) (*79*) with the humpback whale variant file using BCFtools merge to infer the ancestral state. Variants in the humpback whales were deemed ancestral if the variant was observed at a high frequency (>95%) and identified in the fin or/and blue whale genome sequence. An annotation database was created with SnpEff build, using the blue whale reference genome annotation files (NCBI GenBank accession for GFF3 files GCF_009873245.2). The annotation and predicted impact of variants were restricted to canonical variants (-canon), avoiding up or downstream effects (-ud 0). Variant positions with annotation warnings or errors were removed from the analysis.

### Estimation of temporal changes in *N*_E_

Historical changes in *N*_E_ were estimated using the linkage disequilibrium-based approach implemented in GONE (*17*). First, whole-dataset variant files from contemporary samples were converted to map and ped formats using the PLINK recode function, removing sites with missing genotypes (F_MISSING = 0). Main Estimations were performed with a maximum recombination value (*h*_C_) of 0.05. The maximum number of SNPs per chromosome was set to 50,000. The default settings were applied for the remaining parameters (PHASE = 2, cMMb = 1.0, DIST = 1, NGEN = 2000, NBIN = 400, MAF = 0.0, ZERO = 1, maxNCHROM = −99, REPS = 40). Estimates were based on 300 independent runs. Additional *N*_E_ estimates were performed using *h*_C_ thresholds of 0.01 and 0.10 (fig. S4). A lower cutoff value, e.g., 0.01, may improve the resolution in recently admixed populations (*17*). However, in our case, these lower thresholds resulted in a reduced resolution during the most recent generations, likely due to a decrease in SNP density associated with lower thresholds. The mean, median, and 95% CIs were calculated using the dplyr package in R (*82*). The conversion of *N*_E_ estimates to *N*_C_ was conducted assuming an *N*_E_:*N*_C_ ratio of 1:4, as previously used for humpback whales (*34*). We took advantage of the clear drop in *N*_E_ observed in the Southern Ocean 12 to 13 generations ago, which undoubtedly represents the massive decline in humpback whale abundance during the very early phase of the mechanized whaling in Antarctic waters that began in 1904. Using this reference point, we estimated the average generation time to be 8 years. To assess the robustness of the GONE-based *N*_E_ estimates, we calculated *G* for each generational *N*_E_ mean estimate, as suggested by Santiago *et al.* (*17*)

$$G=\frac{n\sqrt{\vartheta }}{N_{E}}$$

where *n* is the sample size and ϑ is the total number of pairwise SNP comparisons used in the estimation.

### Correlation between estimated changes in *N*_E_ and the removal of humpback whales by whaling

Pearson’s correlations were performed using the estimated generational *N*_E_ mean values and the cumulative catches for each of the ocean basins. The catch data were obtained from Smith and Reeves (*83*) in the North Atlantic and from Rocha and colleagues (*18*) in the Southern Ocean. Cumulative catches were summed on the basis of the above generation interval of 8 years. Correlations were performed using Pearson’s correlation sm_statCorr function in the CRAN R package smplot2 (v. 0.2.5) (*84*).

### Sample clustering

Putative genetic structure was assessed for all samples using the unlinked SNPs in the data generated for comparative analyses. Possibly linked SNPs were pruned using the PLINK (v. 1.9) (*85*) indep-pairwise function, with the following parameter settings: 50 SNP windows, 5 SNP steps, and a correlation threshold (*r*^2^) at 0.2. This pruned dataset was used in a principal components analysis (PCA). The PCA was conducted using the smartpca function in EIGENSOFT (v. 8) (*86*). In addition, individual ancestry coefficients were calculated using ADMIXTURE (v. 1.3) (*87*). Admixture proportions and Wright’s fixation index (*F*_ST_) (*88*) were accessed for models ranging from one to five clusters (*K*). For each value of *K*, cross-validation (CV) results were also obtained using 100 bootstrap runs (--CV 100).

### Diversity, inbreeding, and genetic load estimates

Historical genomes where the average read depth was below four were excluded from estimations of heterozygosity, inbreeding, and genetic load (individuals SG36, SG37, SG44, and SG45). Genome-wide heterozygosity (*Het*) was calculated as the ratio of the number of heterozygous genotypes to the total number of callable sites in the individual genome. ROHs were estimated from the temporal SNP datasets using the hidden Markov model implemented in BCFtools roh (*89*). The minimum number of homozygous genotypes per ROH was set at 50 (*-G50*), and the allele frequencies were estimated from the dataset (*-e*). Individual *F*_ROH_ was estimated as the proportion of the autosomal genome located in ROHs longer than 1 Mbp (table S3). The potential and realized mutation load were estimated as the total number of derived variants and the frequency of homozygotes for derived variants, respectively, for each SnpEff impact category (table S4).

The change in variant frequencies in each impact category (*C*) was estimated between the historical (*x*) and contemporary (*y*) samples using the *R*_*x*/*y*_ ratio according to Xue *et al.* (*56*), where

$$R_{x/y}\left(C\right)=\frac{L_{x,y}\left(C\right)}{L_{y,x}\left(C\right)}$$

$$L_{x,y}\left(C\right)=\sum_{x\in C}\;f_{i}^{x}\left(1-f_{i}^{y}\right)$$

$$f_{i}^{x}=\frac{d_{i}^{x}}{n_{i}^{x}}$$

*n_i_* and *d_i_* denote the total and derived number of gene copies in genome position *i*, respectively. *L* denotes the sum of all positions within *C*. Only genome positions with a derived variant were included in the *R*_*x*/*y*_ calculations (*14*, *90*). The variance and 95% CI were estimated by jackknifing sites with a minimum of one derived variant in each category across the autosomal chromosomes.

### Validation of the temporal dataset

Validation of the estimates using the historical genomes was performed using only transversion variants retrieved from the final dataset using the BCFtools view tool. As deamination profiles are mostly found as transition changes at the 5′ and 3′ ends of sequence reads, transversions can serve as a “control” data in ancient DNA studies (*91*, *92*). Analyses were carried out as described above for clustering, genome-wide heterozygosity, ROH, and genetic load estimates, applying the filtered transversions-only dataset.

### Forward simulations

Simulations were also performed on the basis of the demographic parameters estimated in this study, particularly for the Southern Ocean. The SLiM2 v. 4.0 (*57*) framework was used to model population demography and to obtain key statistics on temporal changes. Genome representations were modeled according to the number of genes (*n* = 25,314) found in each chromosome of the blue whale reference genome (*79*). Each gene was simulated as 1000 bp, resulting in a represented genome size of 23 Mbp. The pedigree-based humpback whale mutation rate (1.12 × 10^−8^) was applied (*35*). Selection coefficients of detrimental variants were modeled as in humans (*93*) following Nigenda-Morales *et al.* (*49*). A total of 500,000 burn-in cycles were simulated with a constant *N*_E_ = 16,000, followed by a rapid exponential bottleneck to *N*_E_ = 375 over five generations, with a subsequent recovery to *N*_E_ = 8000 over 10 generations. Key parameter values (heterozygosity, ROHs, and derived detrimental variant counts) were retrieved from the generational cycles by sampling 12 diploid genomes per population in 100 independent simulation replicates.
