# Supplementary material for: Humpback whale genomes reflect the increased efficiency of commercial whaling
Source: Sci Adv. 2025 Dec 17;11(51):eady7091. doi: 10.1126/sciadv.ady7091 (PMC12710703; doi:10.1126/sciadv.ady7091)
Supplement: Supplementary file 1 — Supplementary Text Figs. S1 to S9 Tables S1 to S5 [file sciadv.ady7091_sm.pdf]

Science Advances
AAAS

Supplementary Materials for

Humpback whale genomes reflect the increased efficiency of commercial whaling

Fabricio Furni *et al.*

Corresponding author: Fabricio Furni, fabriciofurni@palaeome.org; Per J. Palsbøll, palsboll@gmail.com

Sci. Adv. **11**, eady7091 (2025)
DOI: 10.1126/sciadv.ady7091

This PDF file includes:

Supplementary Text
Figs. S1 to S9
Tables S1 to S5

Provenance

We generated sequences of 16 genomes from contemporary skin biopsies collected in the North Atlantic and Southern Ocean (table S2). Nine contemporary skin biopsies from Southern Ocean humpback whales were collected between 2002 and 2007 in the waters off the western Antarctic Peninsula. Seven western North Atlantic humpback whale skin biopsies were collected in the Gulf of Maine between 1992 and 2011. An additional 12 genome sequences were obtained from historical bone samples from the early mechanised industrial whaling period in Grytviken, South Georgia, in which the average endogenous DNA content was 79% (standard deviation (SD) 19%). On average, 94% (SD 4%, table S2) of the autosomal genome was sequenced (i.e., a read depth > 1X) in the historical bone samples. The minimum was 84%. The deamination profile in all historical bone samples was as expected for DNA extracted from historical remains, i.e., between 5 - 15% deamination at the ends of individual reads before deamination filtering (fig. S7). The average read depth in the contemporary North Atlantic and Southern Ocean genome sequences was ~ 29X (SD 0.48X) and ~ 14X (SD 0.78X), respectively. The average read depth in the historical Southern Ocean genome sequences was lower (6X, SD 1.2X). After alignment filtering of small and low-quality reads, mapping qualities were similar or superior for historical samples (mean mapping quality for historical genomes: 58.6 SD 0.19, contemporary genomes: 58.1 SD 0.11, fig. S8). After variant calling and quality filtering, 8396422 and 5126003 SNPs were detected among the contemporary North Atlantic (N=7) and Southern Ocean (N=9) genome sequences, respectively (table S3). Nonetheless, the merged "temporal" dataset comprised 1565852 biallelic SNPs (N=24).

Clustering assessment

We performed PCA and ADMIXTURE clustering based on 382916 unlinked SNPs ($r^2 < 0.2$) as well as 184137 unlinked SNPs comprising transversions only. The first principal component revealed two major clusters, representing the two ocean basins; one containing the North Atlantic samples and the other the contemporary and historical Southern Ocean samples (fig. S4). ADMIXTURE failed to detect structure among the samples (most likely value of K = 1, log-likelihood -7709945.545, cross-validation = 0.66745). However, the admixture proportions revealed some geographical and temporal variation among the samples (fig. S9). For instance, for K = 2, samples from the North Atlantic and Southern Ocean appeared distinct with no admixture. At K = 3, the historical samples from the Southern Ocean were also distinct and non-admixed. Increasing levels of admixture were observed at values of K above 3 between the historic and contemporary samples from the Southern Ocean (fig. S9).

Temporal dataset validation

To validate temporal comparisons, we replicated key analyses using only transversion variants to serve as 'control' since the deamination effect in historical genomes is likely represented by transition (C > T/G > A) substitutions (*91*, *92*). We retrieved 663085 transversions from the final SNP dataset. Overall,

the transversion-based results aligned well with those obtained from the full dataset. As expected with the removal of variants, we observed lower heterozygosity (*He*t) values, but still relatively higher loss ratio (~20%) when comparing contemporary and historical datasets (fig. S5A). While the fraction of the genome in runs of homozygosity (ROH) was slightly higher when using only transversions (table S4, fig. S5B), the overall ROH composition remained very similar to that observed using all variant types. None of the genomes showed $F_{ROH>1Mbp} > 0.07$. Counts of derived variants with predicted detrimental effects followed the same trend. While fewer variants were identified due to the exclusion of transitions, we still observed consistent patterns in mutational load, including more homozygous mildly deleterious variants and no significant differences for highly detrimental variants in contemporary genomes from the Southern Ocean and North Atlantic compared to historical genomes, corroborating the findings using the full dataset (fig. S5C).

Additional Provenance

See tables S1 and S2 for provenance information for historical samples used in the study.

fig. S1. Historical demography estimates using GONE. Different GONE plots for different recombination bin thresholds h(c) of 0.05, 0.10, and 0.01. Lines correspond to a mean value based on 100 independent runs. 95% confidence intervals are depicted in shaded areas. Left and right panels: Estimates based on the contemporary Southern Ocean and North Atlantic genomes, respectively.

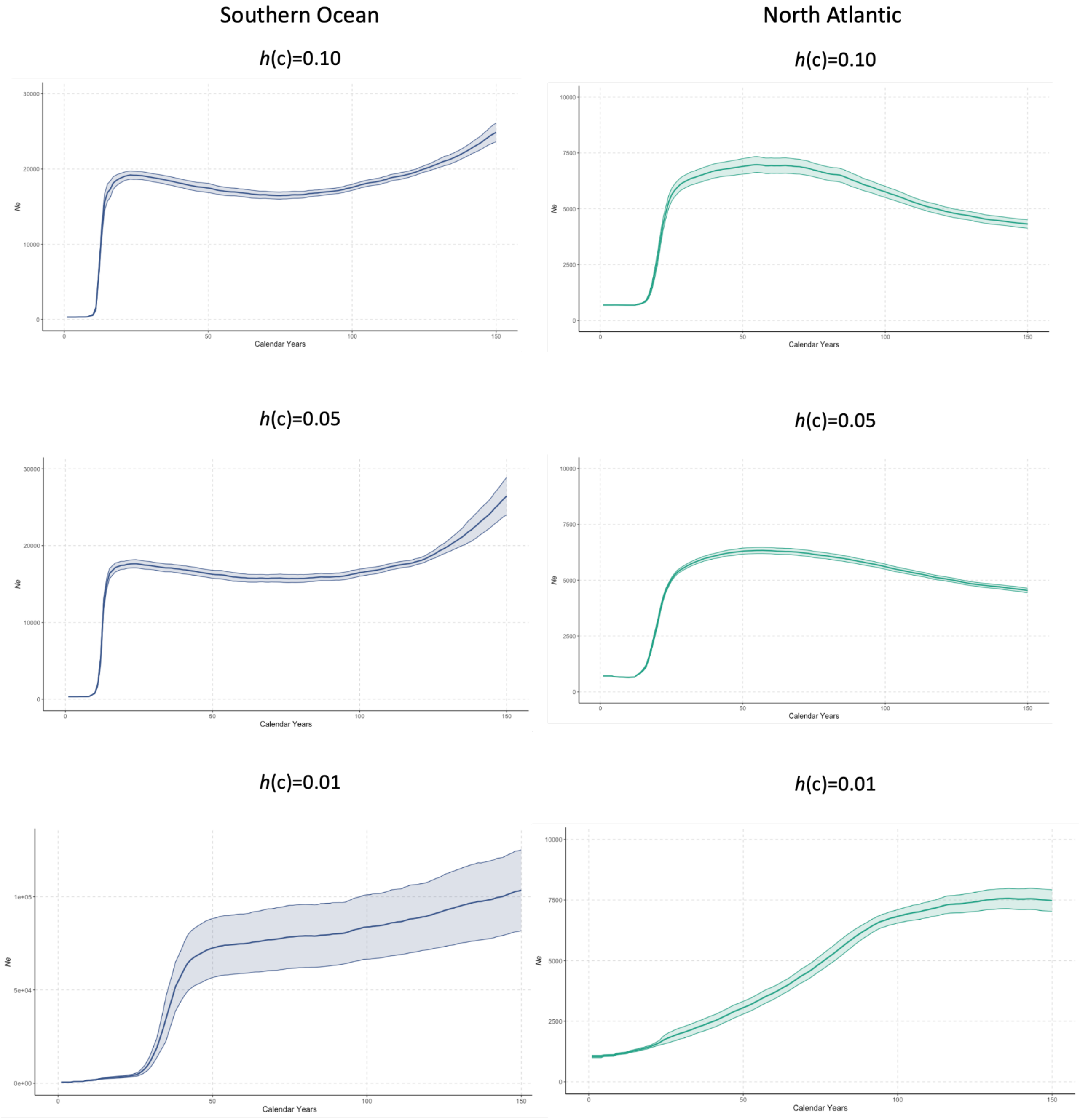

fig. S2. Robustness of the GONE estimates. *G* estimates for each generation (green and blue lines). The red lines denote the *G* threshold (>10) for more robust estimates (*9*). All estimates from 150 generations ago showed *G* > 10, whereas more recent estimates showed *G* > 100.

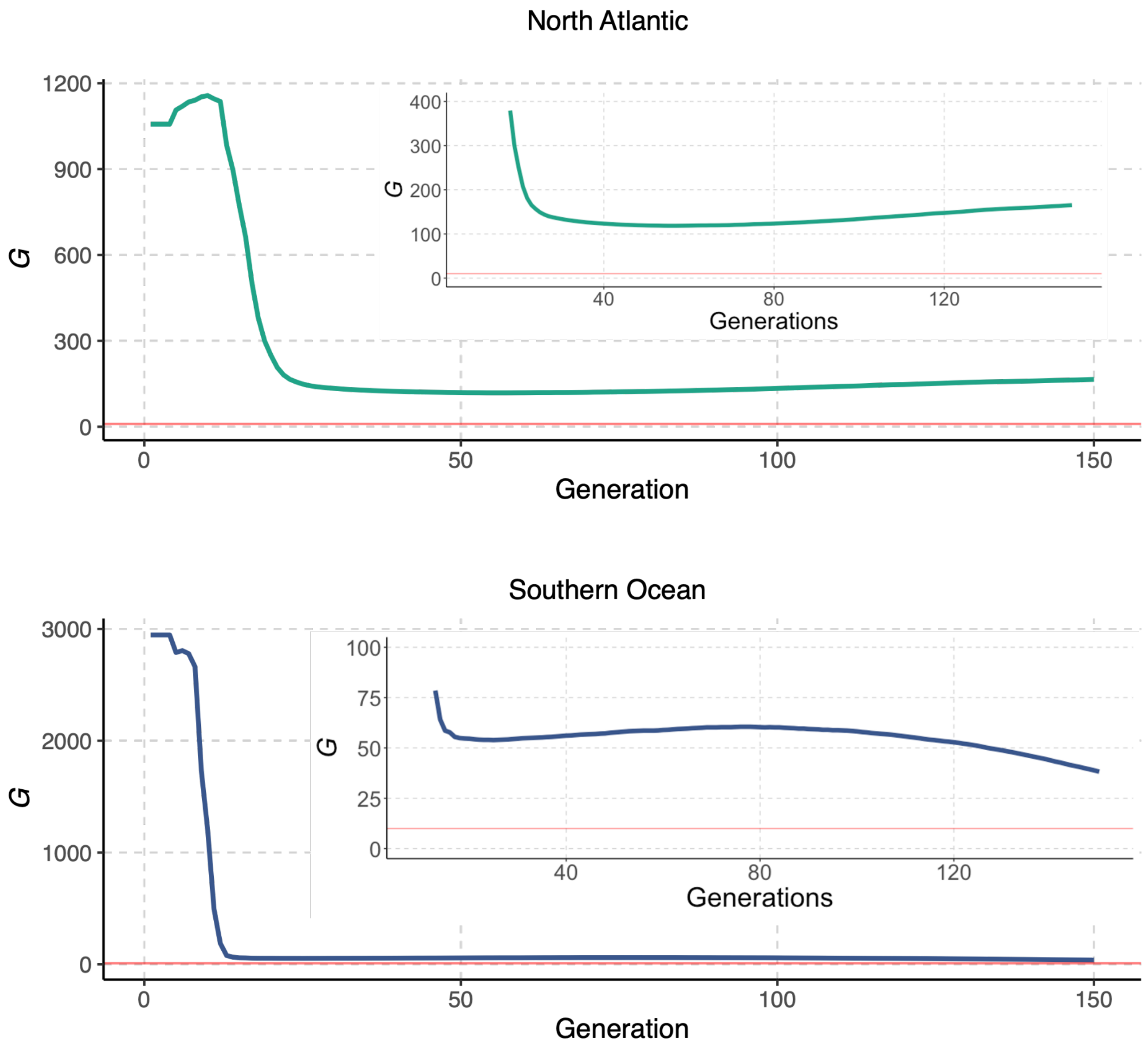

fig. S3. Pearson's correlation between the estimated change in N_E and cumulative removals of humpback whales by whaling. The dashed lines show the fitted regression indicating the direction of the relationship between changes in N_E and cumulative whaling removals.

North Atlantic

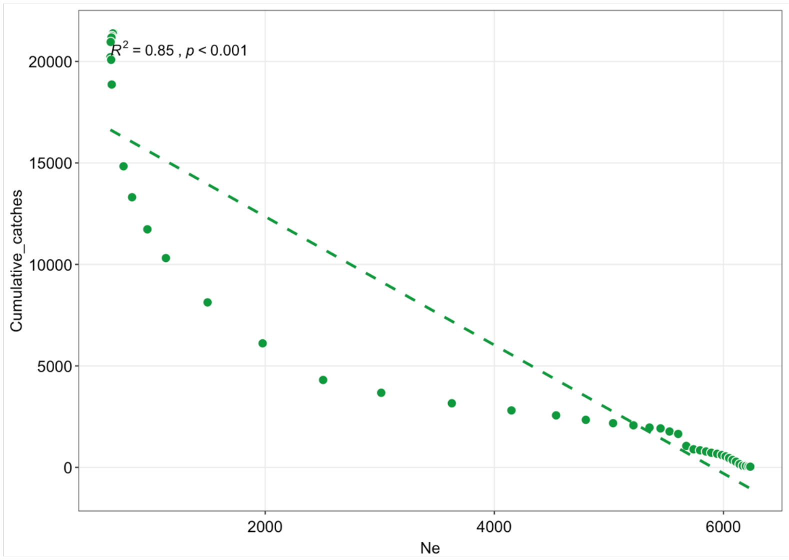

Southern Ocean

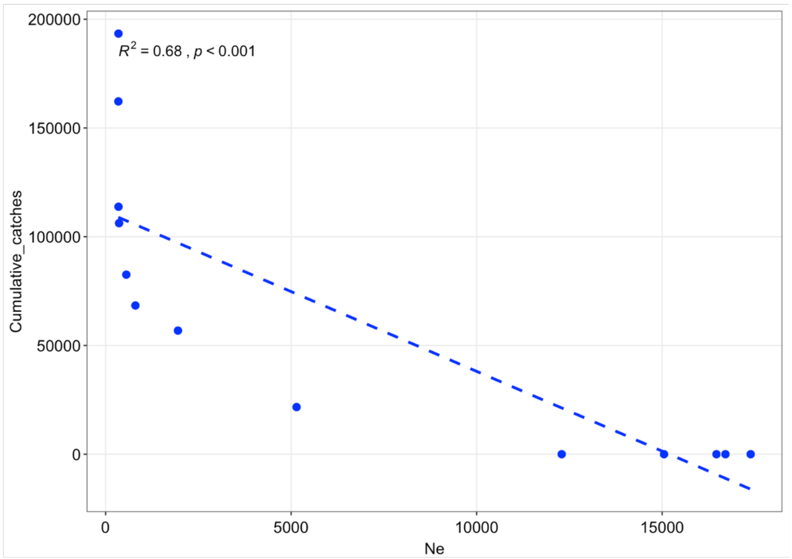

fig. S4. Sample clustering using Principal Component Analysis (PCA). Percentage of the principal component variance in parentheses. Top panel: PCA results using the pruned dataset. Bottom panel: PCA results using the pruned transversions-only dataset. The colours green, blue, and brown indicate contemporary North Atlantic, contemporary Southern Ocean, and historical Southern Ocean samples, respectively. The outgroup was removed from the PCA.

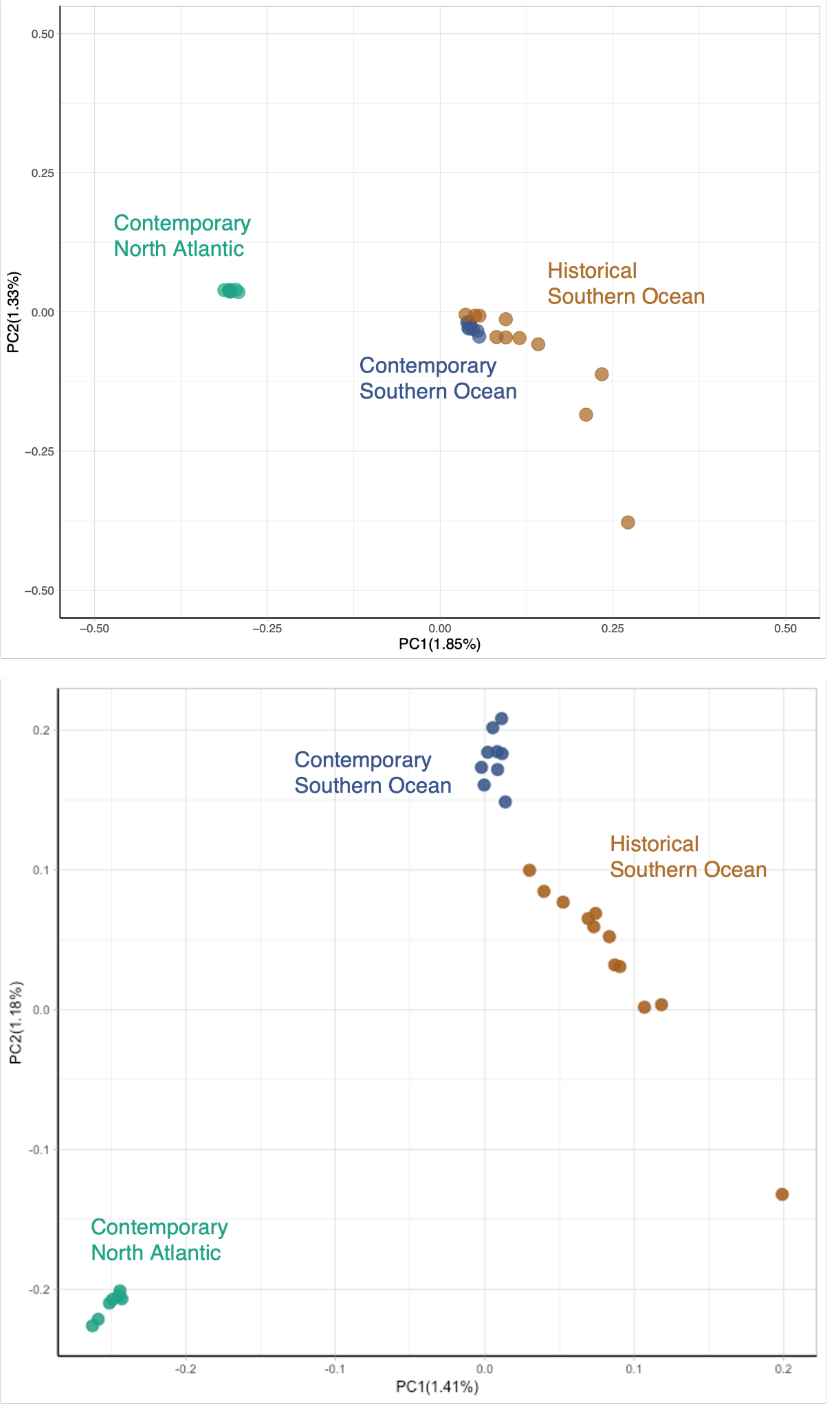

fig S5. Transversions-only results for heterozygosity, runs of homozygosity, and genetic load estimates. **A)** Genome-wide heterozygosity (*Het*) estimates using only transversion variants. **B)** $F_{ROH>1Mbp}$ estimates for transversions only. **C)** Genetic load estimates per impact category for transversion variants only. The estimates obtained from the historical Southern Ocean genomes (HIS), contemporary Southern Ocean (SOU), and the western North Atlantic (NAT) are depicted in brown, blue, and green, respectively.

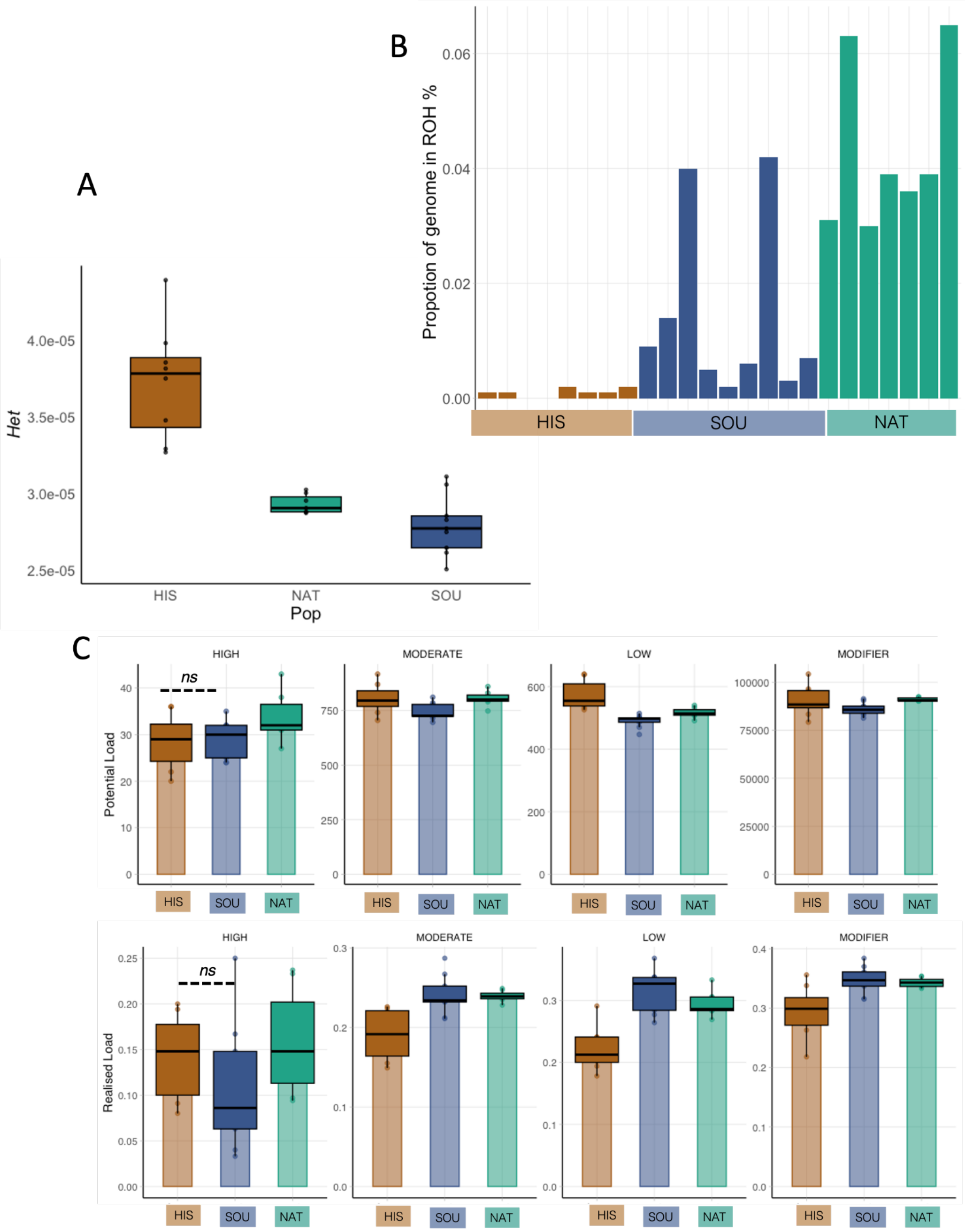

fig. S6. SLiM forward simulations results. Genetic parameters simulated: heterozygosity (*Het*), high realised load, moderated realised load, modifier realised load, and inbreeding coefficients ($F_{ROH>1Mbp}$). Left panel: Mean (black line) and 95% confidence interval (shadowed areas) values based on 100 simulation replicates. The vertical red line indicates the commencement of the bottleneck. Right panel: estimated values retrieved before (10 generations before the bottleneck event) and after the bottleneck (10 generations after the simulation reached the lowest N_E).

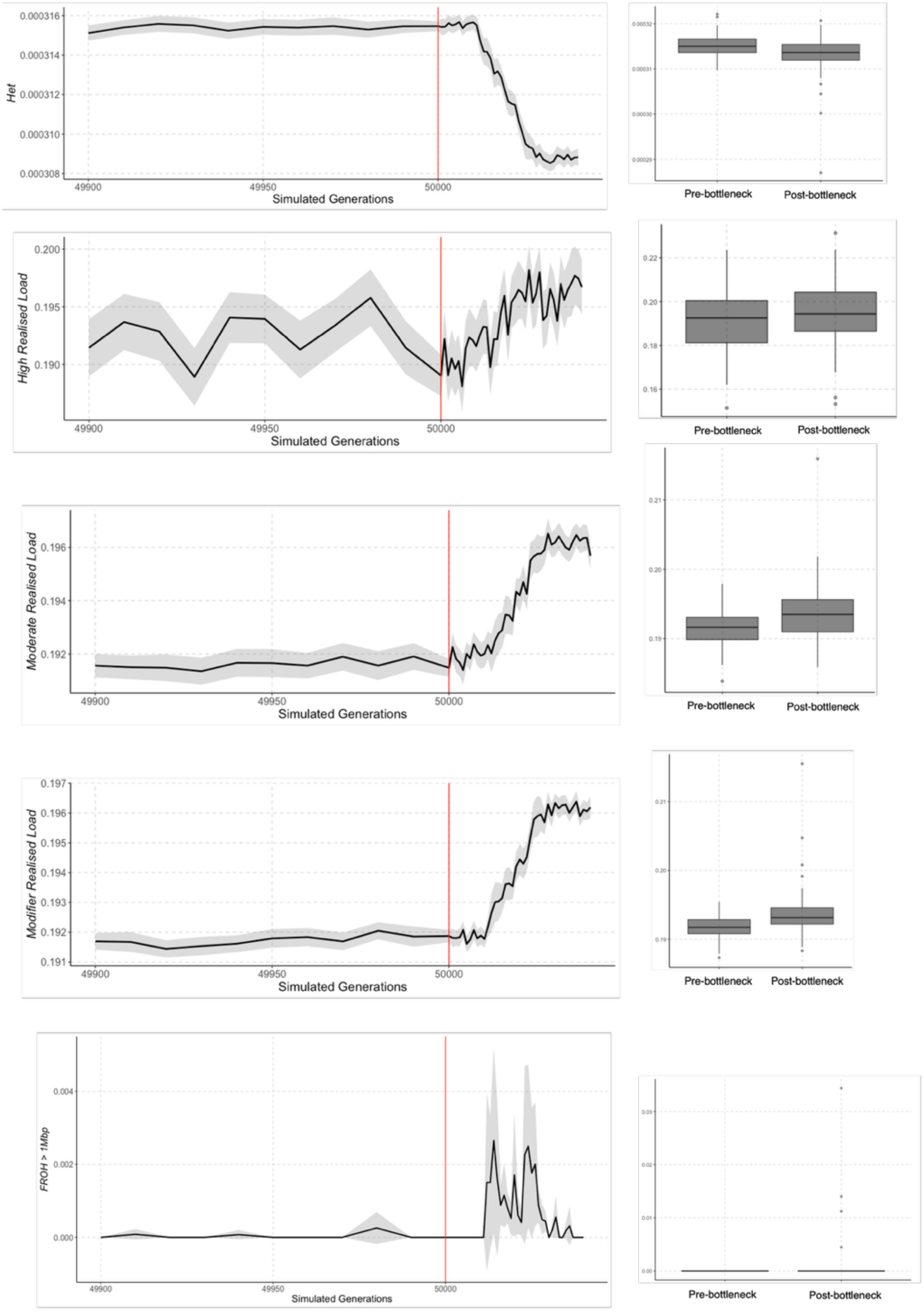

fig. S7. DNA damage frequencies at each end of the raw reads in the historical Southern Ocean samples. Red lines: C to T substitutions, blue lines: G to A substitutions, grey lines: all other substitutions, orange lines: soft-clipped bases.

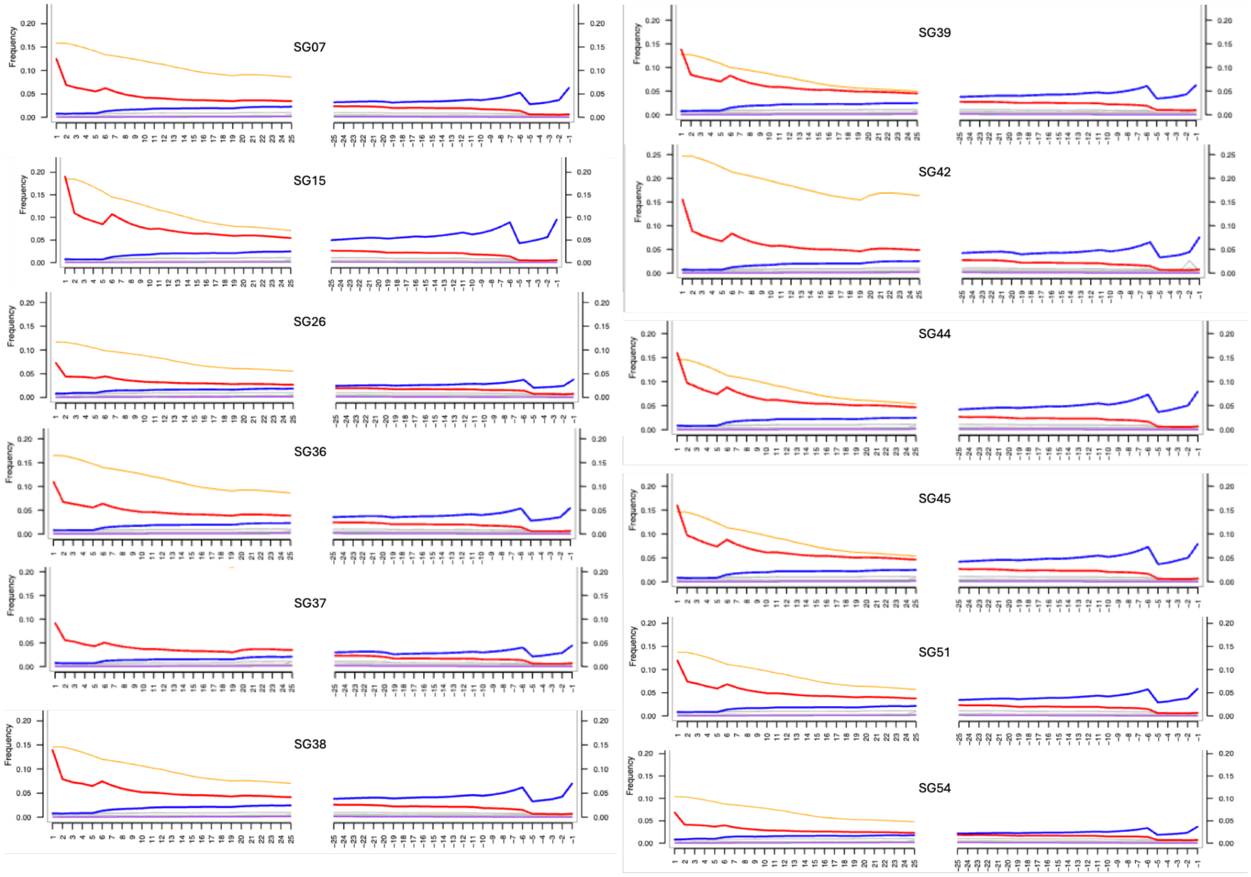

fig S8. Quality metrics for genomes used in the study. A) Average and standard deviation of read sizes for individual genomes. **B**) Average and standard deviation mapping quality for individual genomes. **C**) Distribution of the mapping quality for contemporary and historical genomes. Contemporary samples are denoted in brown and historical samples are denoted in yellow colours.

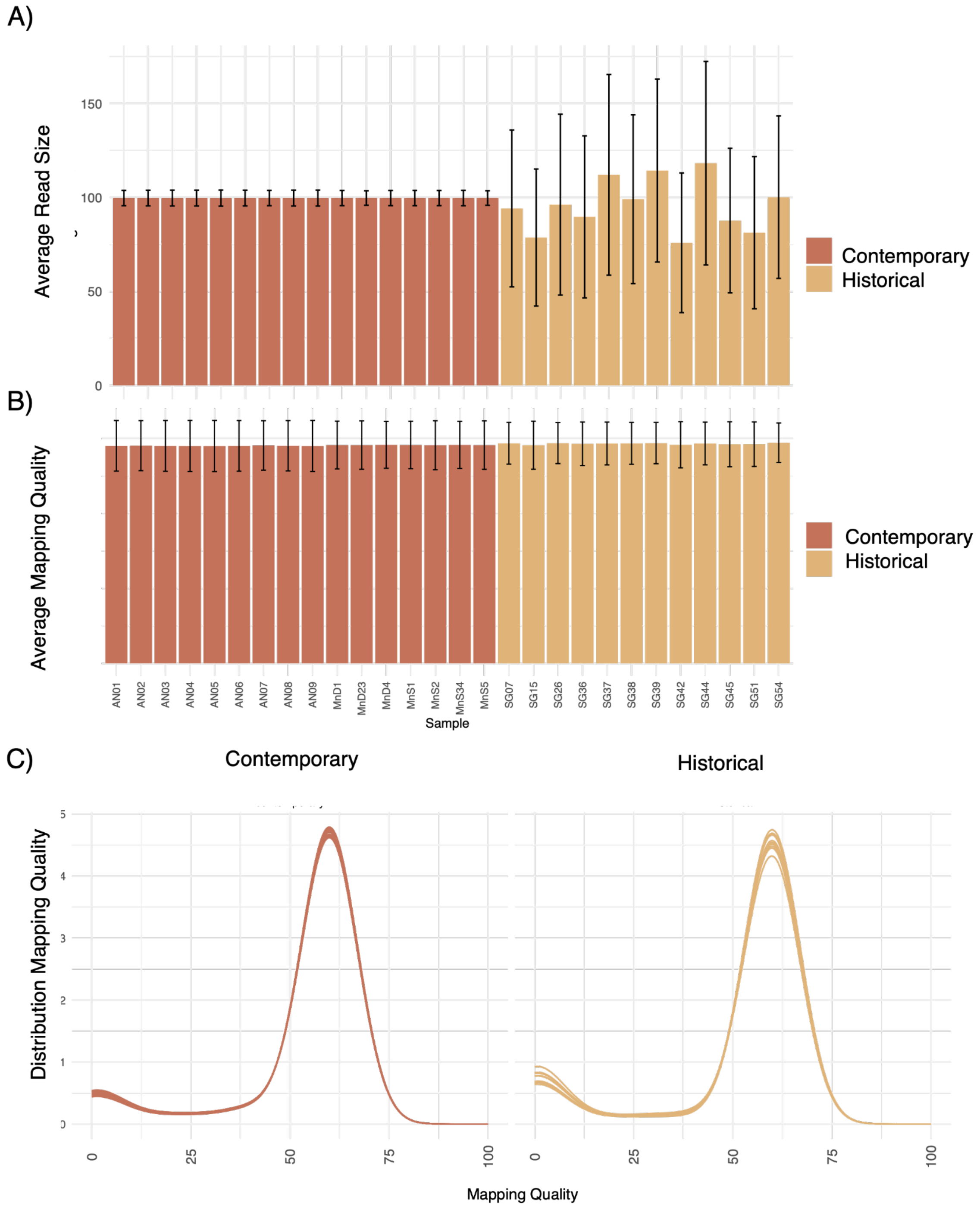

fig. S9. Admixture proportions. Admixture calculated for each individual genome for K = 2 - 5

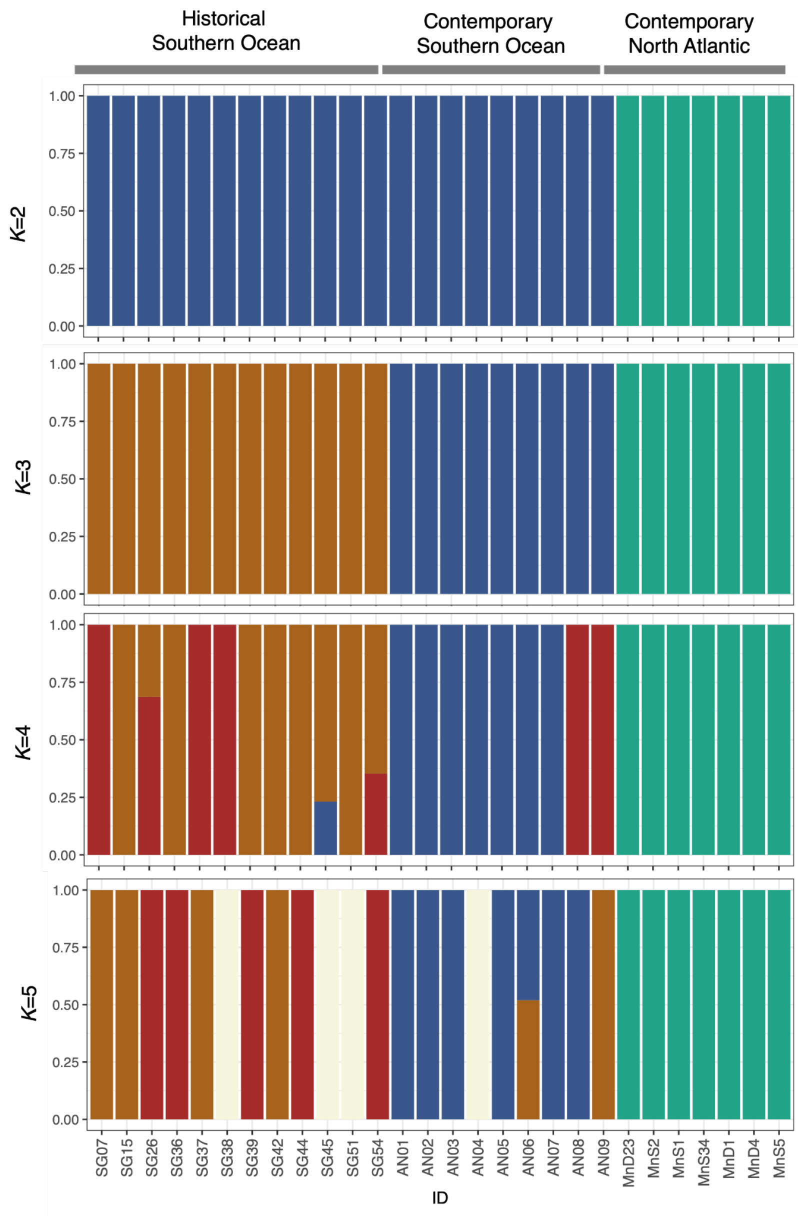

Table S1. Provenance. Information of historical samples provenance used in this study.

Sample	Type	Location	Collected by	Year collected	Species ID	ID Validation	Deposited in
SG07	bone remain	Grytviken, South Georgia Island	Gordon M. Liddle - Government of South Georgia and South Sandwich Islands	2001	ZooMS	Genetics	Mar&Con Group, RUG, Netherlands
SG15	bone remain	Grytviken, South Georgia Island	Gordon M. Liddle - Government of South Georgia and South Sandwich Islands	2001	ZooMS	Genetics	Mar&Con Group, RUG, Netherlands
SG26	bone remain	Grytviken, South Georgia Island	Gordon M. Liddle - Government of South Georgia and South Sandwich Islands	2001	ZooMS	Genetics	Mar&Con Group, RUG, Netherlands
SG36	bone remain	Grytviken, South Georgia Island	Gordon M. Liddle - Government of South Georgia and South Sandwich Islands	2001	ZooMS	Genetics	Mar&Con Group, RUG, Netherlands
SG37	bone remain	Grytviken, South Georgia Island	Gordon M. Liddle - Government of South Georgia and South Sandwich Islands	2001	ZooMS	Genetics	Mar&Con Group, RUG, Netherlands
SG38	bone remain	Grytviken, South Georgia Island	Gordon M. Liddle - Government of South Georgia and South Sandwich Islands	2001	ZooMS	Genetics	Mar&Con Group, RUG, Netherlands
SG39	bone remain	Grytviken, South Georgia Island	Gordon M. Liddle - Government of South Georgia and South Sandwich Islands	2001	ZooMS	Genetics	Mar&Con Group, RUG, Netherlands
SG42	bone remain	Grytviken, South Georgia Island	Gordon M. Liddle - Government of South Georgia and South Sandwich Islands	2001	ZooMS	Genetics	Mar&Con Group, RUG, Netherlands
SG44	bone remain	Grytviken, South Georgia Island	Gordon M. Liddle - Government of South Georgia and South Sandwich Islands	2001	ZooMS	Genetics	Mar&Con Group, RUG, Netherlands
SG45	bone remain	Grytviken, South Georgia Island	Gordon M. Liddle - Government of South Georgia and South Sandwich Islands	2001	ZooMS	Genetics	Mar&Con Group, RUG, Netherlands
SG51	bone remain	Grytviken, South Georgia Island	Gordon M. Liddle - Government of South Georgia and South Sandwich Islands	2001	ZooMS	Genetics	Mar&Con Group, RUG, Netherlands
SG54	bone remain	Grytviken, South Georgia Island	Gordon M. Liddle - Government of South Georgia and South Sandwich Islands	2001	ZooMS	Genetics	Mar&Con Group, RUG, Netherlands

Table S2. Sample information. Sample location, period, and sequencing metrics.

Sample information			Genome sequence				Downsampling	
**ID**	**Location**	**Epoch**	**Endogenous DNA (%)**	**Fraction of duplicate reads**	**Final read depth (X)**	**% genome coverage >1X**	**Fraction**	**Final read depth (X)**
AN01	Southern Ocean	Contemporary	97.5	0.077	12	0.97	0.4	6
AN02	Southern Ocean	Contemporary	97.4	0.062	13	0.97	0.4	6
AN03	Southern Ocean	Contemporary	97.5	0.068	12	0.94	0.4	6
AN04	Southern Ocean	Contemporary	97.6	0.10	14	0.97	0.4	6
AN05	Southern Ocean	Contemporary	97.5	0.086	13	0.94	0.4	6
AN06	Southern Ocean	Contemporary	97.6	0.094	13	0.94	0.4	6
AN07	Southern Ocean	Contemporary	97.5	0.060	12	0.94	0.4	6
AN08	Southern Ocean	Contemporary	97.6	0.082	14	0.97	0.4	7
AN09	Southern Ocean	Contemporary	97.5	0.090	13	0.97	0.4	6
MnD23	North Atlantic	Contemporary	98.7	0.11	29	0.94	0.3	5
MnS2	North Atlantic	Contemporary	98.6	0.12	29	0.97	0.3	5
MnS1	North Atlantic	Contemporary	98.7	0.13	30	0.97	0.3	5
MnS34	North Atlantic	Contemporary	98.6	0.12	29	0.97	0.3	5

Sample information			Genome sequence				Downsampling	
**ID**	**Location**	**Epoch**	**Endogenous DNA (%)**	**Fraction of duplicate reads**	**Final read depth (X)**	**% genome coverage >1X**	**Fraction**	**Final read depth (X)**
MnD1	North Atlantic	Contemporary	98.6	0.13	30	0.97	0.3	5
MnD4	North Atlantic	Contemporary	98.5	0.13	29	0.94	0.3	5
MnS5	North Atlantic	Contemporary	98.7	0.13	29	0.97	0.3	4
SG07	Southern Ocean	Historical	85.5	0.36	5	0.89	NA	5
SG15	Southern Ocean	Historical	92.1	0.47	5	0.87	NA	5
SG26	Southern Ocean	Historical	92.2	0.34	6	0.93	NA	6
SG36	Southern Ocean	Historical	85.2	0.40	4	0.85	NA	4
SG37	Southern Ocean	Historical	67.7	0.53	4	0.86	NA	4
SG38	Southern Ocean	Historical	88.4	0.38	5	0.92	NA	5
SG39	Southern Ocean	Historical	97.0	0.38	6	0.92	-	6
SG42	Southern Ocean	Historical	93.0	0.33	6	0.89	NA	6
SG44	Southern Ocean	Historical	71.8	0.53	3	0.83	NA	3
SG45	Southern Ocean	Historical	95.5	0.33	4	0.82	NA	4

Sample information			Genome sequence				Downsampling	
**ID**	**Location**	**Epoch**	**Endogenous DNA (%)**	**Fraction of duplicate reads**	**Final read depth (X)**	**% genome coverage >1X**	**Fraction**	**Final read depth (X)**
SG51	Southern Ocean	Historical	91.6	0.39	5	0.87	NA	5
SG54	Southern Ocean	Historical	94.7	0.40	7	0.93	NA	7

Table S3. Single-nucleotide polymorphism (SNP) information. The whole dataset includes SNPs obtained from all reads. The downsampled dataset includes SNPs after the downsampling of reads to similar values as historical samples.

Sample ID	Callable sites	Entire dataset		Down-sampled dataset	
		SNPs before filtering	SNPs after filtering	SNPs before filtering	SNPs after filtering
AN01	2107072387	31209514	2341214	25032014	567274
AN02	2115905780	31273552	2343726	25425585	572580
AN03	2103022970	31157055	2321244	24620019	559539
AN04	2121625723	31335590	2343274	25607624	572259
AN05	2133551272	31419609	2352255	25983469	575164
AN06	2129220101	31370970	2345263	25653111	569934
AN07	2121552034	31232366	2318158	25335797	566885
AN08	2139461751	31477672	2350030	26413425	582607
AN09	2144213563	31267709	2343250	26610501	584169
MnD23	2143939572	29519902	4220678	26442111	590260
MnS2	2130008851	29487569	4246591	26183906	591183
MnS1	2147320446	29538574	4260351	26645005	592088
MnS34	2142276787	29509733	4266420	26656091	595956
MnD1	2152837055	29513102	4240066	26701330	592184
MnD4	2143087102	29516990	4261072	26592873	594286
MnS5	2134902567	29471919	4225681	26309594	592234
SG07	1859310633	19693697	555842	19693697	555842
SG15	1776180025	18364115	545320	18364115	545320
SG26	2010108555	24072355	589727	24072355	589727
SG36	1729970658	15531446	512651	15531446	512651
SG37	1781689419	17435031	529598	17435031	529598
SG38	1933349221	22173258	571107	22173258	571107
SG39	1966053070	24451869	595579	24451869	595579
SG42	1854129859	20661822	567584	20661822	567584
SG44	1706500511	14755609	502908	14755609	502908
SG45	1661558367	15773716	526199	15773716	526199

Sample ID	Callable sites	Entire dataset		Down-sampled dataset	
		SNPs before filtering	SNPs after filtering	SNPs before filtering	SNPs after filtering
SG51	1804348900	19960095	568422	19960095	568422
SG54	2019425926	25225937	612150	25225937	612150

Table S4. Genome-wide heterozygosity and runs of homozygosity (ROH) estimates. ROHs larger than 1 Mbps were considered in the calculations. Calculations were performed for all variants and transversions only.

Sample	Heterozygosity	Heterozygosity (transversions only)	Total length of segments in ROH > 1Mbp	$F_{ROH\ (>1Mbp)}$	$F_{ROH\ (>1Mbp)}$ (transversions only)
AN01	7.25E-05	2.65E-05	11905637	0.006	0.0093
AN02	7.50E-05	2.77E-05	21777676	0.010	0.0138
AN03	6.72E-05	2.51E-05	64792717	0.031	0.0401
AN04	7.52E-05	2.83E-05	5575409	0.003	0.0048
AN05	7.65E-05	2.85E-05	1123404	0.001	0.0021
AN06	7.31E-05	2.75E-05	4985311	0.002	0.0063
AN07	7.02E-05	2.61E-05	74823008	0.035	0.0419
AN08	8.20E-05	3.11E-05	1067033	0.0005	0.0029
AN09	8.22E-05	3.06E-05	9386795	0.004	0.0072
MnD23	8.02E-05	2.91E-05	39059481	0.018	0.0314
MnS2	7.96E-05	2.88E-05	29871061	0.014	0.0634
MnS1	8.11E-05	2.95E-05	48997690	0.023	0.0301
MnS34	8.34E-05	3.03E-05	38193943	0.018	0.0392
MnD1	8.02E-05	2.88E-05	90595396	0.043	0.0357
MnD4	8.24E-05	3.00E-05	43226839	0.020	0.0388
MnS5	7.95E-05	2.87E-05	104401001	0.049	0.0650
SG07	9.88E-05	3.29E-05	600131	0.00073	0.0007

SG15	10.5E-05	3.27E-05	621927	0.00099	0.0010
SG26	11.0E-05	3.86E-05	891398	0.00039	0.0004
SG38	10.7E-05	3.48E-05	935899	0.00041	0.0004
SG39	12.3E-05	3.98E-05	1309033	0.00237	0.0024
SG42	11.8E-05	3.75E-05	1049191	0.00046	0.0005
SG51	11.7E-05	3.82E-05	1281077	0.00100	0.0010
SG54	12.5E-05	4.39E-05	2827654	0.00196	0.0020

Table S5. Genetic load information. Absolute counts for total and derived variants based on each of the SNPEFF impact category.

Sample	HIGH				MODERATE				LOW				MODIFIER			
	Total		Derived		Total		Derived		Total		Derived		Total		Derived	
	hom	*het*	*hom*	*het*	*hom*	*het*	*hom*	*het*	*hom*	*het*	*hom*	*het*	*hom*	*het*	*hom*	*het*
AN01	31	46	7	45	1947	1286	504	1247	3513	1241	809	1192	355016	137337	94487	133355
AN02	30	51	7	50	1924	1372	492	1322	3496	1376	819	1313	354431	142699	93879	138529
AN03	33	51	10	49	1955	1302	525	1256	3494	1318	802	1256	358455	126790	97833	122935
AN04	31	52	7	51	1946	1364	495	1327	3535	1330	837	1273	353160	143528	92798	139357
AN05	32	61	7	60	1899	1481	462	1425	3448	1496	759	1430	352657	146743	92092	142604
AN06	28	53	4	51	1969	1391	527	1351	3457	1380	770	1322	354645	140076	94235	135802
AN07	27	47	3	45	1959	1361	517	1325	3496	1251	795	1208	358201	133748	97558	129821
AN08	31	44	7	41	1896	1526	470	1464	3420	1526	741	1445	348108	157698	89944	151065
AN09	29	57	5	55	1917	1509	492	1445	3430	1525	746	1451	349169	158333	90083	152563
MnD23	34	53	10	51	1980	1482	546	1433	3544	1525	847	1470	357983	155070	97177	150879
MnS2	32	54	7	53	2004	1443	561	1391	3522	1524	823	1467	361776	151822	100912	147644

MnS1	34	56	10	55	1960	1453	518	1409	3519	1469	826	1411	358114	156438	97358	152099
MnS34	41	60	17	59	1975	1539	548	1483	3501	1605	802	1542	357692	159956	97304	155208
MnD1	36	54	12	53	1998	1466	558	1425	3543	1521	846	1467	359551	155178	98748	151015
MnD4	37	62	13	61	1952	1566	521	1510	3469	1571	779	1514	358071	158141	97450	153686
MnS5	37	64	13	62	1981	1518	547	1463	3552	1542	861	1484	362274	152338	101512	147996
SG07	28	49	8	44	1731	1500	454	1309	3043	1717	662	1360	316893	164820	82362	135293
